# Influence of very low doses of mediators on fungal laccase activity - nonlinearity beyond imagination

**DOI:** 10.1186/1753-4631-3-10

**Published:** 2009-09-04

**Authors:** Elzbieta Malarczyk, Janina Kochmanska-Rdest, Anna Jarosz-Wilkolazka

**Affiliations:** 1 Biochemistry Department, Maria Curie-Skłodowska University, Lublin, Poland

## Abstract

Laccase, an enzyme responsible for aerobic transformations of natural phenolics, in industrial applications requires the presence of low-molecular substances known as mediators, which accelerate oxidation processes. However, the use of mediators is limited by their toxicity and the high costs of exploitation. The activation of extracellular laccase in growing fungal culture with highly diluted mediators, ABTS and HBT is described. Two high laccase-producing fungal strains, *Trametes versicolor *and *Cerrena unicolor*, were used in this study as a source of enzyme. Selected dilutions of the mediators significantly increased the activity of extracellular laccase during 14 days of cultivation what was distinctly visible in PAGE technique and in colorimetric tests. The same mediator dilutions increased demethylation properties of laccase, which was demonstrated during incubation of enzyme with veratric acid. It was established that the activation effect was assigned to specific dilutions of mediators. Our dose-response dilution process smoothly passes into the range of action of homeopathic dilutions and is of interest for homeopaths.

## Background

Laccases (EC 1.10.3.2, p-diphenol: dioxygen oxidoreductases) are multi-copper proteins that use molecular oxygen to oxidize various phenolic and non-phenolic aromatic compounds by a radical-catalyzed reaction mechanism. Laccases can also recognize as glycoproteins since carbohydrates take part in the chemical stabilization of enzymatic molecules [1,2]. Fungi produce laccases for degradation of polymeric phenolic compounds which are the main source of carbon. Molecule of laccase is too large for direct contact with the phenolics present in the inner part of such biopolymers as lignin or the lignocellulose complex, so the process of degradation runs very slowly and needs to cooperate with specific co-catalizators. They can enhance laccase activity by using low molecular compounds which accelerate the catalytic properties of enzyme by improving their affinity of specific radical forms to phenolic biopolymers [3]. Fungal laccases, as well known biocatalysts, are readily used in many branches of industrial technology. Among them the depolymerization [4] and biobleaching of lignin [5], decolorization of artificial dyes in the textile industry [1,6] or of natural pigments in food technology [7] could be mentioned. Laccases can also oxidize xenobiotic compounds [8] and remove of lipophilic compounds from paper pulp [9]. Another important activity of laccase is demethylation of non-phenolic lignins [10,11]. At the beginning of this process, non-phenolic lignin particles, rich in methoxylic compounds as veratrate or anisate, are degraded by laccase to phenolic compounds mainly via demethylation and hydroxylation and these processes are accelerated in combination with redox co-catalysts known as mediators [12,13]. Many popular mediators of laccase are recognized also among the natural products of lignin and humus degradation and syringaldehyde, acetosyringone, vanillic, p-coumaric and ferulic acids can be mentioned [14,15]. These natural mediators represent an alternative to synthetic mediators which are more efficient but unfortunately more toxic for environment and more expensive in exploitation.

Commonly used synthetic laccase mediators belong to phenolics. Most of them contain *N*-hydroxy-groups as 1-hydroxybenzotriazole (HBT), 2,2'-azino-bis(3-ethylbenzthiazoline-6-sulphonic acid) (ABTS), and *N*-hydroxy-acetanilide (NHA) or contain pirimidine origin such as violuric acid (VA), which can disturb the organization of nucleic acids [16,17]. Also transition metal complexes, such as K_4_Mo(CN)_8 _or K_4_W(CN)_8_), were suggested as a new class of laccase mediators for pulp bleaching [18,19]. For industrial purposes, both synthetic and metal-complex mediators must be used in large quantities, which is not very safe for the environment and is very costly. To diminish these difficulties, various methods have been tested and among them, electrochemical methods [20,21] and production of laccase recombinants could be mentioned. In our earlier papers, the opportunity of using the low doses of laccase effectors during fungal cultivation was tested [22]. The possibility to enhance laccase efficiency by the incubation with high dilutions of synthetic mediators is now presented. These experiments are based on our earlier results with changes of plant peroxidase activity in the presence of very low doses of selected phenolics [23,24]. The oscillating character of the mentioned changes allowed us to indicate the dilutions of the tested aromatic substances which reveal a maximum and a minimum effect on enzymatic activity. These changes had been tested using spectrophotometrical and luminometrical methods [25]. In the present study the technique of electrophoresis (PAGE) of native extracellular laccase from cultures of *T. versicolor *and *C. unicolor *was used to compare of isozymic patterns of laccase growing with or without low doses of mediators, ABTS and HBT.

## Methods

### Biological material

Two species of white rot fungi with a high index of extracellular-laccase production, *Trametes versicolor *(L, ex Fr.) Pil and *Cerrena unicolor *(Bull. Ex Fr.) Murr, [26-28] originated from the culture collection of the Department of Biochemistry of Maria Curie-Sklodowska University in Lublin were used in the study.

### Preparation of mediator dilutions

The substances affecting the activation of fungal laccase were chosen from among mediators well-known from the literature. These were ABTS and HBT, the both at an initial concentration of 1 mol/l. Each mediator was subjected to dynamic dilution in 75% ethanol by successive transfer to new portion of the diluent at 1:100 ratios. The Eppendorf tubes, every fulfilled with 1,98 ml of 75% ethanol, were shaken with 10 vertical strokes after serial transfer of 0,02 ml of mediator dilution according to homeopathic manner [23-25]. The dilutions were numbered in accordance with the number of transfers so that n = 1 corresponded to the one-hundredth dilution of the stock solution, n = 2 was a one-hundredth dilution of solution n = 1, etc. The transfers were usually repeated 10 times to obtain the final solution with n = 10 at a concentration of 100^-10 ^mol/l. In the same manner the successive dilutions of 75% ethanol were prepared for control purpose. For comparative studies, dilution series which required 31 successive transfers (iterations) were prepared for ABTS.

### Fungal cultivation

Stock cultures grew stationary in liquid medium according to earlier report [23]. In these conditions fungal mycelium is growing in the shape of flat comb. The common used technique with transfer of mycelia disks from stock cultures [29] were used for production of parallel cultures for comparison an action of serial dilutions. Mycelial discs were punched out with a 7 mm diameter cork borer after 10 days of cultivation from the stock culture mycelium under sterile conditions and every 3 discs were placed in flasks containing 10 ml of fresh medium. Starting from the second day of cultivation, each culture was supplemented every two days with 200 μl of serial dilution of ABTS and HBT, prepared as described in the above manner. The control cultures were lead with 200 μl of serial dilutions of 75% ethanol, puted in the same days as dilutions of mediators for dividing the effect of ethanol and mediators [30]. To average the results, three cultures were performed for each dilution. The cultures were grown in stationary mood for fourteen days. After that time, the media were separated and the activity of extracellular laccase was measured.

### Laccase activity assay

Laccase activity was measured spectrophotometrically using syringaldazine (2.5 μM) as the substrate in 0.1 M citrate-phosphate buffer, pH 5.2 according to the method [31]. The activity was expressed as nkatals/l, using molar absorption coefficients of 65,000 M^-1 ^cm^-1 ^at 525 nm.

### Non-denaturing polyacrylamide gel electrophoresis

PAGE technique with 12% poliacrylamide slabs without SDS was used to compare the native laccase isozyme patterns. For electrophoretic test the 5 ml volume of every medium after the end of cultivation was used according to [32]. The media samples were demineralized on the column with Sephadex G25, concentrated by ultrafiltration using Microcon Centrifugal Filter Units, 3000 NMWL (Millipore) and lyophylised. After dissolution, 25 μl of every sample were deposited on 9-well slab, and the routine electrophoresis was run. After finishing of experiment the slabs were immediately specifically stained for laccase activity and enzymatic bands were visualized by the rapid color reaction with guaiacol in 0.1 M citrate-phosphate buffer at 25°C and pH 4.8 [33].

### Veratrate demethylation assay

Laccase from *T. versicolor *was purified in routine manner according to [34].

**Figure 1:**
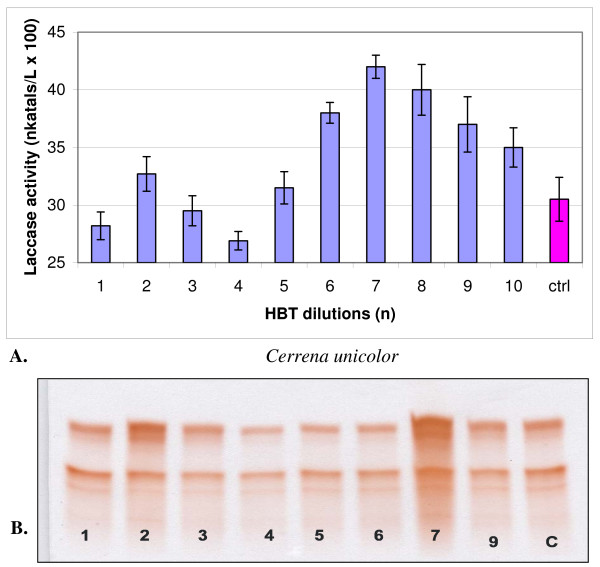
**Changes in the activity (A) and isozymic patterns (B) of extracellular laccase in media of *Cerrena unicolor *cultivated 14 days with 10 dilutions of HBT**. On PAGE slab (B) the dilutions No 8 was omitted; c (ctrl) - control. PAGE slab stained with guaiacol.

**Figure 2:**
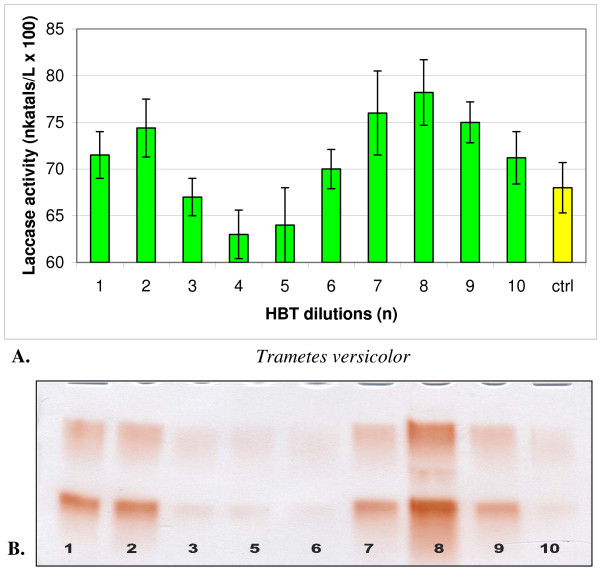
**Changes in the activity (A) and isozymic patterns (B) of extracellular laccase in media of *Trametes versicolor *cultivated 14 days with 10 dilutions of HBT**. On PAGE slab (B) the dilutions No 4 was omitted; ctrl - control PAGE slab stained with guaiacol.

**Figure 3:**
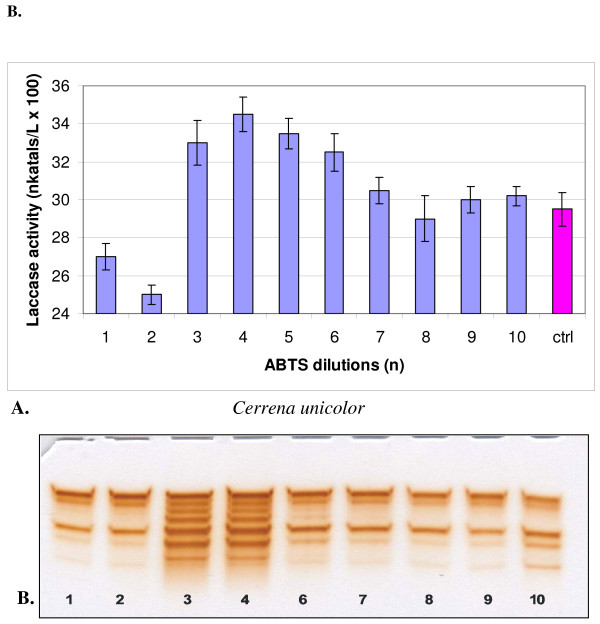
**Changes in the activity (A) and isozymic patterns (B) of extracellular laccase in media of *Cerrena unicolor *cultivated 14 days with 10 dilutions of ABTS**. On PAGE slab (B) the dilutions No 5 was omitted; ctrl - control. PAGE slab stained with guaiacol.

**Figure 4:**
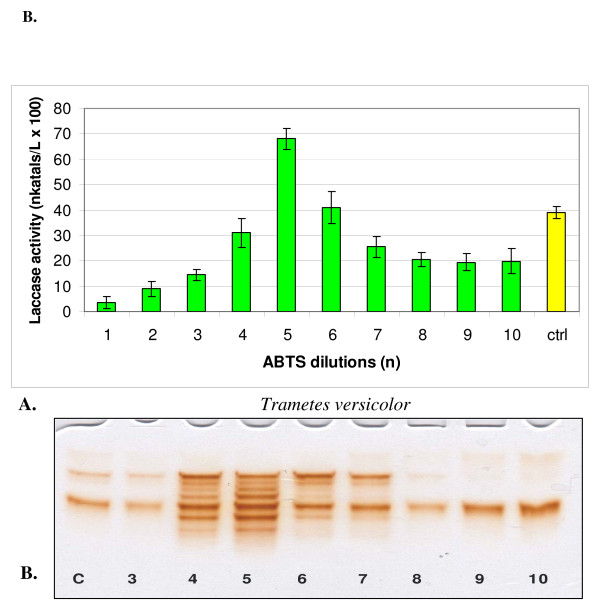
**Changes in the activity (A) and isozymic patterns (B) of extracellular laccase in media of *Trametes versicolor *cultivated 14 days with 10 dilutions of ABTS**. On PAGE slab (B) the dilutions No 1 and 2 was omitted; c (ctrl) - control. PAGE slab stained with guaiacol.

Two milliliters of 14 days culture media or pure laccase solution with the beginning activity 20 nkatals/ml were incubated with one ml of 2% potasium veratrate during 4 hours in the presence of 200 μl selected dilutions of HBT or ABTS, chosen according to PAGE results from Fig 1, 2, 3, 4, 5. The selected dilutions of HBT (n = 5 and 11) and ABTS (n = 6 and 12) were used respectively according to their maximal and minimal action on the laccase activity. The progress of veratrate demethylation was assayed spectrophotometrically based on a colorimetric reaction of vanillates [35] which are the main product of veratrate demethylation. After incubation, the rapid colorimetric reaction with DASA reagents was run and 0.2 ml of each sample was mixed with 0.2 ml of 2% sulphanilamide solution in 10% HCl followed by addition of 0.2 ml of 5% NaNO_3_. The mixture was neutralized with 1 ml of 20% Na_2_CO_3_, and absorbance at 500 nm was measured. The % of progress in veratrate demethylation was calculated with the calibration curve for vanillic acids (y = 6.85x- 0.0128, R^2 ^= 0,999).

**Figure 5:**
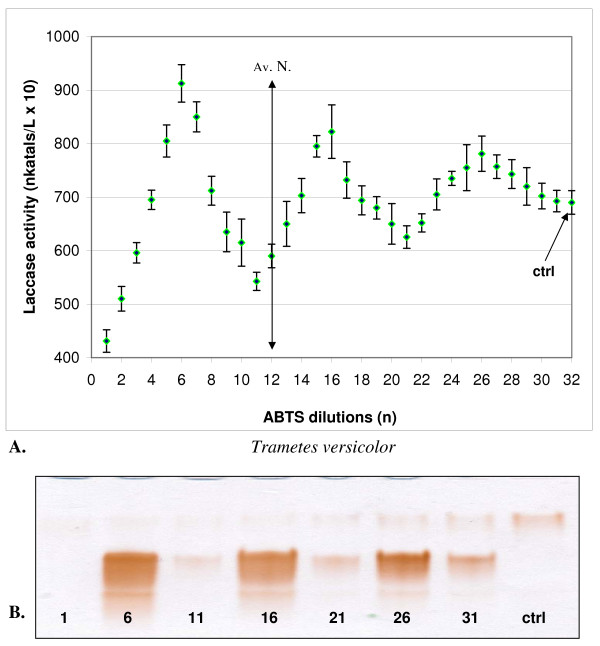
**Changes in laccase activity (A) in media of *Trametes versicolor *cultivated 14 days with 31 dilutions of ABTS and isozymic patterns (B) for chosen dilutions of mediator**. The vertical line corresponds to Avogadro number; ctrl -- control. PAGE slab stained with guaiacol.

## Results

### 1. Changes in laccase activity in cultures of *T. versicolor* and *C. unicolor* in the presence of low doses of mediators

Fungal strains were grown for 14 days with the addition of the mediators, ABTS and HBT, diluted serially in 75% ethanol as described in Methods. Each culture was treated every two days with a new portion of 200 μl of a given dilution. It can be mentioned that although after 14 days of culture the mycelium growth was similar for all trials, including the control cultures, but the activity of extracellular laccase was different, depending on the type of mediator and the degree of its dilution. The curves of laccase activities had always sine shape, which were characteristic for the type of mediator and kind of fungi.

In cultures of both strains, one-hundredth dilutions of HBT in a series from 1 to 10 showed two maxima and one minimum (Figures 1 and 2). A maximum common to both fungi appeared at dilution n = 2, which corresponded to the concentration of 100^-2 ^mol/l, and additionally at n = 7 for *C. unicolor *(100^-7 ^mol/l) and n = 8 for *T. versicolor *(100^-8 ^mol/l). ABTS showed maximum activation of laccase at dilution n = 4 in the case of *C. unicolor *and at n = 5 in the case of *T. versicolor *(Figures 3 and 4). Generally, the values of activation were lower in *C. unicolor*, than in *T. versicolor *(Table 1). A comparison of the shape of curves for two fungal strains showed that the maxima of activity towards ABTS occurred at opposite positions to the points of activity towards HBT.

**Table 1:** Extracellular laccase activity (Δ max - min) in media of 3 series of *Trametes versicolor *cultures and 3 series of *Cerrena unicolor *cultures growing 14 days in presence of dilutions of ABTS and HBT with extreme action on laccase activity (in katals/l).

***Trametes versicolor***			***Cerrena unicolor***		
**ABTS**	**HBT**	**ABTS/HBT**	**ABTS**	**HBT**	**ABTS/HBT**

6500 ± 150	1550 ± 92	4.2 ± 1.6	950 ± 65	275 ± 45	3.4 ± 1.4
6200 ± 120	1410 ± 79	4.4 ± 1.51	1220 ± 83	370 ± 72	3.3 ± 1.15
5900 ± 180	1311 ± 103	4.5 ± 1.74	980 ± 75	280 ± 55	3.5 ± 1.36

For *Trametes versicolor*: ABTS (Δ between nominal values for dilutions 5 and 1), HBT (Δ 8-4), *Cerrena unicolor*: ABTS (Δ 4 -- 2), HBT (Δ 7 - 4).

The electrophoretic patterns, prepared in the same manner for all variants, confirmed changes in the intensity of enzymatically active bands parallel to sine curve of enzymatic activity visible after specific guaiacol staining for laccase activity. A particular distinctive change occurred in cultures activated with ABTS, where all seven laccase isozymes underwent a significant intensification (Figures 3 and 4). The next experiments were lead only with ABTS dilutions in cultures of *T. versicolor *for the higher results then with *C. unicolor* cultures and HBT.

With series of ABTS dilutions extended to 31 iterations the next uncommon observations in changes of laccase activity were obtained. After crossing the value of Avogadro number (dilution with n = 12) the differences in activity were visible in agreement with sine curve shape. It was also confirmed by electrophoretic patterns. The amplitude of maximal and minimal values showed the declining character (Figure 5)

### 2. The influence of selected mediator dilutions on the demethylation activity of purified laccase

**Figure 6:**
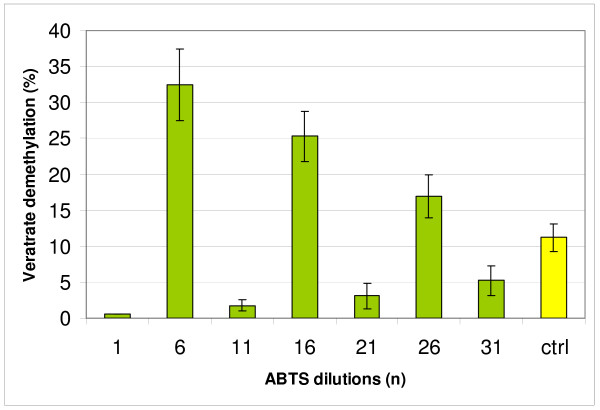
**Demethylation of veratrate with media after 14 day cultivation of *Trametes versicolor *incubated 4 hours with extreme dilutions of ABTS chosen according to PAGE results on Figure 5; ctrl - control**.

To characterize influence of different mediator dilutions on laccase activity the test with veratrate demethylation was done. In the first series of experiment with ABTS and *T. versicolor*, the abilities of chosen dilutions to initiate demethylation process were observed during the common incubation of the culture media, veratrate and proper mediator dilution (Figure 6). Because the generally low speedy of demethylation process [36] the time of four hours of incubation was chosen. The amount of demethylation products (mainly two isomeric vanillic acids) was determined spectrophotometrically with DASA reaction. The regular changes in demethylation activity were visible shaped like sine curve also for dilutions greater than the Avogadro number.

In the next part of studies on demethylation two extreme dilutions for the both mediators were chosen based on the results of experiments with PAGE (Figures 1, 2, 3, and 4), and incubation with pure laccase and veratrate was lead as above mentioned. The dilutions with maximal activity in changing the isozymic patterns were also very active in the demethylation processes.

**Figure 7:**
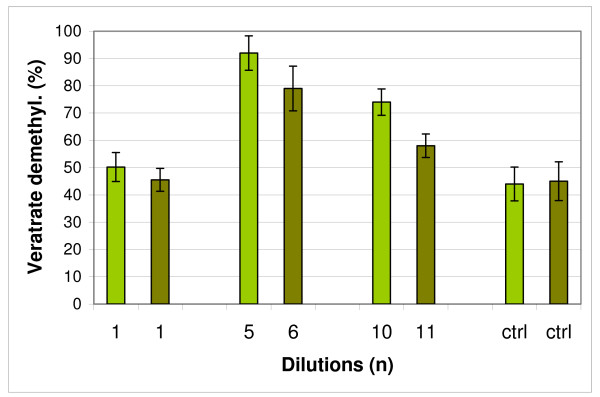
**Demethylation of veratrate with pure laccase from *Trametes versicolor *incubated 4 hours with three extreme dilutions of ABTS (light green) and HBT (dark green) chosen according to PAGE results (see Figures 2 and 4); ctrl - control**.

Even the dilutions with an inhibitory effect against laccase activity (Figure 7) showed *in vivo *higher demethylation action than control samples. The results presented on Figure 7 showed that the same dilutions of mediators which were active *in vivo *for changing isozymic patterns and enzymatic activity also modify accordingly demethylating abilities of laccase in experiments *in vitro*.

These results confirmed that isozymic patterns, demethylation activities and activation/deactivation processes were influenced differently by mediators of different dilutions *n*, shaped like sine curve.

## Discussion

The presented results of activation or inhibition of extracellular laccase in laboratory cultures under the influence of diluted synthetic mediators ABTS and HBT are in accordance with the action of the law of hormesis. This law describes the opposing biological effect of diluted effectors and concerns regulation of the speed of biological response to a given stimulus based on the phenomenon of feedback and allosteric properties of complex protein systems [37]. During gradual dilution, the intensity of a biological effect oscillates in a sinusoidal manner in accordance with a characteristic hormetic curve, which changes dynamically in time. The universality of the oscillatory influence of high dilutions on processes taking place in human and animal organisms has been pointed out in a growing number of studies, for instance those cataloged in the years 2001-2008 by Calabrese [37,38].

In the experiments on fungal laccase, studies of the influence of the individual mediator dilutions in the order in which they had been prepared made it possible to distinguish those which activated laccase from those which inhibited it, and the use of two fungal species additionally showed that each of them had an individual pattern of the sine curve. This is an important observation, from which it follows that the results obtained in the present study cannot be put directly into practice without constructing appropriate hormetic curves in the conditions of a given laboratory. Also, there are yet other species of industrial fungi that need to be studied in which selective use of highly diluted mediators may strengthen the ability to produce laccase. The studies presented here show that among the molar dilutions, a clearly activating effect was exerted by those of the order of picomoles, corresponding to the 100^-6 ^mol/l concentration of the mediator. Use of picogram, instead of gram amounts of a mediator in industry may lead to a significant reduction of industrial-scale use of mediators without lowering the effects of their action, which is promising not only on account of the general reduction of production costs but also because of a decreased pollution of the environment. It can also be mentioned that diluted mediators may act on fungal cultures without additional sterilization procedures if high percent ethanol is used in preparing the dilutions. These dependencies allow applying experimentally selected dilutions of fungal laccase mediators for intensification of oxidation processes during transformation of laccase substrates such as phenols and methoxyphenols.

The process of incubation of high-molecular-weight lignin polymers with laccase applied in the paper industry is long-lasting and its supplementation with diluted effectors may have a practical use in shortening the contact of wood with a fungus. In accordance with the results shown in Fig. 6, an activating concentration of ABTS which corresponds to dilution n = 6 is within the range of 100^-6 ^mol/l and is thus a picogram quantity. Even repeated supplementation with such a strongly-diluted effector will remain safe for the environment and will considerably decrease the costs of applying these useful laccase-activating compounds.

It seems that this remains true for both native laccase from *in vitro *cultures and for the pure enzyme, after its isolation and purification. PAGE data indicate that the effect of the diluted effectors reaches as far as the molecular level of cells, increasing the processes of isozyme production in the fungal cell. A well-visible strengthening of the bands of laccase activity, corresponding to the process of intensification of laccase induction by the most active dilutions, dependent on the type of mediator and kind of fungal strain, was observed during our study. We also noted a simultaneous augmentation of the pool of laccase secreted extracellularly from cells to the culture medium.

Although the double action of biologically active substances, dependent on the degree of dilution, has been confirmed in numerous experiments, this phenomenon has not received a complete explanation within the accepted scientific canons. Currently, more and more studies point to the possibility of explaining the biological effect of low doses by means of physical and chemical instruments [39-43]. Probably, in the future it will be possible to use those instruments to explain the phenomenon, described in this work, of reaction continued for dilutions greater than the Avogadro number. This phenomenon was already signaled earlier in the 1980s by French researchers [44] for human and animal organisms and has now also been demonstrated for plant material [45]. Also similar results with fungal material or pure enzymes were presented earlier by us, and in all those experiments the conformity and repeatability of the results were very high. All these data seem to constitute a prelude to recognizing hormesis, so far considered in molecular categories, to be an initial stage of the dose-response dilution process which smoothly passes into the range of action of homeopathic dilutions greater than the Avogadro number. Although the phenomenon has not yet been explained by contemporary science (cf. [46]), the growing number of common observations enforces the need to acknowledge the facts and the possibility of their practical implementation just as unfamiliarity with the scientific basis of fermentation did not prevent the Ancients from consuming large amounts of wine. The physico-chemical character of the discussed phenomenon seems important for the biology and biotechnology of fungal laccase because of its practical consequences in reducing the toxic effect of industrially applied laccase mediators on the environment.

## Conclusion

During cultivation of two fungal strains, *Trametes versicolor *and *Cerrena unicolor*, in the presence of high diluted laccase mediators (ABTS, HBT), changes in the production and activity of extracellular laccase in the culture medium were observed. The profile of the activity had the character of a sine curve with distinct maxima and minima. These changes were also noticed in the enzymatic patterns after specific visualization of laccase activity on PAGE gel, and the correlation between maximum activities on the sine curve and the abundance of laccase-active protein bands was very distinct. The selected dilutions of the mediators caused similar changes in the demethylation abilities of pure laccase isolated from *T. versicolor*. All these results strongly bear out the new features of diluted substances against selected enzymes tested in *in vitro *and *in vivo *conditions. The biphasic role of high diluted low molecular effectors on the laccase system of fungal cells is best understood as an instance of hormesis, in which every substance after dilution can act *in vivo *either as an inhibitor or as an activator of a biological process dependent on the rate of dilution. As for laccase applications, the possibility of radically diminishing the amount of its mediators in industrial processes with simultaneous augmentation of its production during fungal cultivation is very real. For this reason, the mentioned results are very interesting both from the economic point of view and from the perspective of environmental protection against chemical pollution.

The presented study should be treated mainly as a report on the newly discovered ability of selected dilutions of commonly used mediators to change the activity of laccase. Practical application of these results in industrial processes will require from factories adaptation of this knowledge to their own experimental conditions.

## Competing interests

The authors declare that they have no competing interests.

## Authors' contributions

EM conceived of the study, and participated in its design and coordination. JKR carried out the PAGE assay and coordinate experiments. AJW participated in the sequence alignment. All authors read and approved the final manuscript.
